# Defense Suppression through Interplant Communication Depends on the Attacking Herbivore Species

**DOI:** 10.1007/s10886-021-01314-6

**Published:** 2021-09-20

**Authors:** Laura O. Marmolejo, Morgan N. Thompson, Anjel M. Helms

**Affiliations:** Department of Entomology, Texas A&M University, College Station, TX 77843-2475 USA

**Keywords:** Herbivore-induced plant volatiles, Defense suppression, Defense priming, Herbivore resistance, Plant defense

## Abstract

In response to herbivory, plants emit volatile compounds that play important roles in plant defense. Herbivore-induced plant volatiles (HIPVs) can deter herbivores, recruit natural enemies, and warn other plants of possible herbivore attack. Following HIPV detection, neighboring plants often respond by enhancing their anti-herbivore defenses, but a recent study found that herbivores can manipulate HIPV-interplant communication for their own benefit and suppress defenses in neighboring plants. Herbivores induce species-specific blends of HIPVs and how these different blends affect the specificity of plant defense responses remains unclear. Here we assessed how HIPVs from zucchini plants (*Cucurbita pepo*) challenged with different herbivore species affect resistance in neighboring plants. Volatile “emitter” plants were damaged by one of three herbivore species: saltmarsh caterpillars (*Estigmene acrea*), squash bugs (*Anasa tristis*), or striped cucumber beetles (*Acalymma vittatum*), or were left as undamaged controls. Neighboring “receiver” plants were exposed to HIPVs or control volatiles and then challenged by the associated herbivore species. As measures of plant resistance, we quantified herbivore feeding damage and defense-related phytohormones in receivers. We found that the three herbivore species induced different HIPV blends from squash plants. HIPVs induced by saltmarsh caterpillars suppressed defenses in receivers, leading to greater herbivory and lower defense induction compared to controls. In contrast, HIPVs induced by cucumber beetles and squash bugs did not affect plant resistance to subsequent herbivory in receivers. Our study shows that herbivore species identity affects volatile-mediated interplant communication in zucchini, revealing a new example of herbivore defense suppression through volatile cues.

**Supplementary Information:**

The online version contains supplementary material available at 10.1007/s10886-021-01314-6.

## Introduction

Although immobile, plants are not defenseless and instead use a wide variety of strategies to protect themselves against insect herbivores. Herbivore feeding or presence on a plant triggers the production of volatile compounds (Helms et al. 2014; Pashalidou et al. 2015), and these herbivore-induced plant volatiles (HIPVs) serve important defensive functions—directly repelling herbivores (Bernasconi et al. 1998; De Moraes et al. 2001), intoxicating herbivores (Veyrat et al. 2016), and attracting natural enemies that kill herbivores (Clavijo Mccormick et al. 2014; Grunseich et al. 2020). The compositions of HIPV blends are typically specific to the identity of the plant and the attacking herbivore species, conveying detailed information to other members of the ecological community, including natural enemies (Clavijo McCormick et al. 2012), other herbivores (Ray et al. 2020), and even other plants (Arimura et al. 2009). A growing number of studies have reported that plants can detect HIPVs from herbivore-damaged neighbors as a warning of possible herbivory and respond by enhancing their defenses (Karban et al. 2014). Here, we examine the specificity of plant responses to HIPVs by exposing plants to species-specific HIPV blends induced by three different herbivore attackers and quantifying plant defense activation.

Induced defenses against herbivores are hypothesized to allow plants to efficiently allocate resources and activate effective defenses against specific attackers only when needed (Mithöfer and Boland 2012; Schultz et al. 2013; Karban 2020). Previous studies have documented remarkable specificity in plant responses to different herbivore species (Ali and Agrawal 2012; Bonaventure 2018). Plants detect herbivory though cues associated with their own damaged tissues (Tanaka and Heil 2021), as well as specific herbivore-associated cues (Acevedo et al. 2015). Through these recognition systems, plants differentiate between herbivores with different feeding strategies or guilds—for example, chewing herbivores that cut and remove plant tissue or piercing-sucking herbivores, with comparatively less physical damage, that penetrate plant tissues to feed on phloem or xylem (Rodriguez-Saona et al. 2010; Davidson-Lowe et al. 2019). Generally, chewing and piercing-sucking herbivores induce different plant defenses and corresponding signaling molecules, or phytohormones, as chewing herbivory often induces jasmonic acid (JA) and piercing-sucking herbivory induces salicylic acid (SA) (Erb et al. 2012). Signaling antagonism, or crosstalk, between JA and SA is often observed (Thaler et al. 2012), indicating defense against a particular herbivore can induce susceptibility to a different attacker. Despite these broad patterns associated with plant defense against herbivory, different species of herbivores within the same feeding guild can also trigger distinct plant responses (De Moraes et al. 1998; Chung and Felton 2011; Sobhy et al. 2017). Herbivores can also fall under two extreme categories of host-plant range: specialists that feed on plants within one family, or generalists that feed on a wide range of plants from multiple families. Herbivore dietary specialization can further shape plant defenses, as generalists are often more susceptible to toxic plant chemicals compared to specialists that have coevolved with these toxins and may require different defense strategies (Ali and Agrawal 2012). Based on their sophisticated detection systems, plants rely on common defensive strategies for broad classes of herbivores but can also fine-tune inducible defenses to specific attackers.

In addition to induced defenses, plants can further optimize their defense investment strategies through priming. Priming is the physiological process by which a plant prepares to more quickly or aggressively respond to future biotic or abiotic stresses, typically conferring plants with enhanced resistance (Martinez-Medina et al. 2016). Chemical cues associated with herbivory, including herbivore pheromones (Helms et al. 2013) and HIPVs from neighboring plants (Grof-Tisza et al. 2020; Pashalidou et al. 2020) can prime plant defenses, enhancing defense induction in response to herbivore attack. A limited number of plant volatile compounds have been identified as plant priming cues, including several green leaf volatiles (Engelberth et al. 2004), methyl jasmonate (Karban et al. 2000) and indole (Erb et al. 2015). Notably, these compounds are conserved cues present in the HIPV blends for many plant–herbivore species combinations, indicating ubiquitous volatiles may prime a wide variety of plant species (Karban et al. 2003; Kessler et al. 2006; Zakir et al. 2013). Recent findings support the idea of low specificity in plant responses to HIPVs, as tobacco plants exposed to HIPVs induced by two caterpillar species, *Manduca sexta* and *Heliothis virescens*, showed similar priming responses when challenged by either herbivore species (Paudel Timilsena et al. 2020). In contrast, other research suggests HIPV specificity can play important roles in interplant communication, such as sagebrush plants (Asteraceae) eliciting stronger priming responses in their kin compared to unrelated plants (Karban et al. 2013) and in local neighbors compared to foreign conspecifics (Karban et al. 2016). Furthermore, another recent study reported herbivore species-specificity in HIPV production and responses in neighboring plants. HIPVs from tomato plants (*Solanum lycopersicum*) damaged by beet armyworm caterpillars (*Spodoptera exigua*) enhanced neighboring plant resistance to herbivory, but HIPVs induced by whitefly (*Bemisia tabaci*) damage suppressed neighboring plant defenses and enhanced whitefly performance (Zhang et al. 2019). Despite these advances, our understanding of how HIPV specificity and attacking herbivore identity modulate plant communication remains limited.

Domesticated plant species in the gourd family (Cucurbitaceae) include several high-value vegetable crops, such as zucchini squash (*Cucurbita pepo* L.). Striped cucumber beetles (*Acalymma vittatum* F.) and squash bugs (*Anasa tristis* DeGeer) are specialist herbivores on cucurbit crops and significant agricultural pests in North America (Doughty et al. 2016; Haber et al. 2021). Cucurbits are also occasionally attacked by generalist herbivore species, including highly polyphagous saltmarsh caterpillars (*Estigmene acrea* Drury) (MNT personal observations). Cucurbit plants produce a class of bitter-tasting triterpenoid defense compounds called cucurbitacins (Da Costa and Jones 1971), and these compounds mediate plant interactions with the herbivore community (Theis et al. 2014). Generalist herbivores are often deterred by cucurbitacins, whereas specialists detoxify or sequester them for protection against predators and may perceive them as phagostimulants (Ferguson and Metcalf 1985). Many domesticated cucurbit varieties, however, lost the ability to produce cucurbitacins or produce only low levels (Theis et al. 2014; Brzozowski et al. 2019), and recent studies have implicated plant volatiles as important chemical cues in cucurbit-herbivore interactions (Shaprio and Mauck 2018). Indeed, cucurbit volatiles play key roles in herbivore host-plant selection (Brzozowski et al. 2020), plant disease transmission by vector herbivores (Mauck et al. 2010a), and natural enemy attraction to host plants to kill herbivores (Agrawal et al. 2002; Grunseich et al. 2020). Nevertheless, few studies have characterized beetle-induced HIPVs in zucchini squash (Brzozowski et al. 2020) and none have examined squash bug- or saltmarsh caterpillar-induced HIPVs in zucchini squash. Further, whether HIPV-mediated interplant communication occurs in Cucurbitaceae remains an unexplored question, which could elucidate critical information for agroecological interactions between plants and herbivores.

The overall goal of this study was to evaluate the specificity of plant volatile communication by characterizing the defense responses of zucchini squash plants exposed to HIPVs from different herbivore species. We characterized the HIPV blends of squash plants attacked by three herbivore species: saltmarsh caterpillars, squash bugs, or striped cucumber beetles. To determine how these HIPV blends influenced plant priming, volatile “emitter” plants were damaged by one of the three herbivore species or were left as undamaged controls. We exposed neighboring “receiver” plants to HIPVs or control volatiles from emitters and then challenged receivers with herbivory by the associated herbivore species. As measures of plant resistance, we quantified the amount of herbivore feeding damage and levels of defense-related phytohormones induced in receiver plants. Based on predicted HIPV production following attack from herbivores of different feeding guilds, we expected squash plants exposed to HIPVs from chewing herbivores (saltmarsh caterpillars and cucumber beetles) would have enhanced resistance to herbivory, while plant exposure to HIPVs from piercing-sucking herbivores (squash bugs) would have no effect on plant resistance. This research highlights additional complexity in plant responses to HIPVs and offers insight into how volatile cues associated with different herbivore species affect plant defense responses.

## Materials & Methods

### Plants and Insects

Zucchini squash plants (*Cucurbita pepo* ssp. *pepo* cv. Raven) were grown from seed (Johnny's Selected Seeds, Fairfield, USA) and used in experiments after 3–4 weeks of growth. Plants were grown in individual 10-cm diameter pots in topsoil mix (Hyponex Corporation, Marysville, USA) with 3 g Osmocote® fertilizer (15–9-12 N-P-K; Scotts, Marysville, USA) and were kept in an insect-free, climate-controlled growth room with supplemental lighting (16 h light: 8 h dark; 22 °C: 29 °C; 56% RH, Fluence, Austin, USA). Saltmarsh caterpillars (*Estigmene acrea*), squash bugs (*Anasa tristis*), and striped cucumber beetles (*Acalymma vittatum*) were maintained in separate laboratory colonies on cultivated cucumber (*Cucumis sativus* cv. Max Pack) and squash (*C. pepo* cv. Raven) plants. All colonies were kept at 25 °C on a 16 h light: 8 h dark schedule in College Station, USA. Caterpillars, squash bugs, and beetles were originally obtained from Hillsboro, College Station, and State College, USA, respectively, and all colonies were periodically supplemented with wild-caught adults collected near College Station, USA.

### Volatile Collection and Analysis

To determine how herbivory by different herbivore species affects zucchini volatile emissions, we characterized volatiles from plants with and without herbivory by saltmarsh caterpillars, squash bug nymphs, or adult cucumber beetles. We used dynamic headspace sampling to collect volatiles emitted by control and herbivore-damaged leaves. Prior to collections, we caged either 4 fifth-instar saltmarsh caterpillars (n = 5)*,* 14 fourth-instar squash bug nymphs (n = 5), or 5 adult cucumber beetles (n = 5) on treatment plants, while control plants (n = 4–5) remained undamaged. The number and developmental stage of each species was selected based on availability and field-relevant herbivore abundances (Singer et al. 2004; Mauck et al. 2010b; Brzozowski et al. 2020). Saltmarsh caterpillars and cucumber beetles fed for 24 h and squash bugs fed for 48 h prior to collections (Fig. S1A). Plants with actively feeding insects or control plants were placed inside individual 4-L glass chambers (Rogers Custom Glass, Warriors Mark, USA). We collected volatiles for 8 h during photophase (14:00–22:00) pushing filtered air at a rate of 2.6 L/min. Simultaneously, air was pulled out of chambers through an absorbent filter (containing 45 mg of HayeSep® Q (Hayes Separations Inc., Bandera, USA) at 1.0 L/min. After 8 h, insects were removed, and plant tissue was collected and dried at 35ºC to calculate the quantity of volatiles per gram of plant tissue.

We eluted the volatile filter traps using 150 μL dichloromethane and added 5 μL of a standard containing nonyl acetate (80 ng/μL) to each sample. Volatiles were analyzed using an Agilent 7890B gas chromatograph and 5977B mass spectrometer with a splitless injector held at 250 °C and helium as the carrier gas. After sample injection (1 μl), the column (HP-5MS 30 m × 0.250 mm-ID, 0.25 μm film thickness; Agilent Technologies, Santa Clara, USA) was held at 40 °C for 5 min before the temperature was increased at 20 °C/min to 250 °C. Compounds were ionized by electron impact ionization at 70 eV and mass spectra were acquired by scanning from 40 to 300 m/z at 5.30 scans/s. Tentative identification of target compounds was achieved by comparison with mass spectral libraries (NIST17 and Adams2 [Allured Publishing Corporation]), and structure assignments were confirmed where possible by comparison of mass spectra and retention times with authentic standards (Grunseich et al. 2020). Compounds were quantified relative to standard concentrations and calculated as ng/g dried leaf mass.

### Plant Volatile Exposure and Herbivore Challenge

In separate experiments, we exposed squash plants to undamaged control plant volatiles or herbivore-induced plant volatiles from one of three different herbivore species: saltmarsh caterpillars, squash bugs, or cucumber beetles. For each experiment, three plants were placed inside an open-top plastic box (66 × 41 × 36 cm) with equal spacing and no physical contact between plants. The center plant was the volatile “emitter”, and the other two plants were volatile “receivers” (Fig. S2). Emitter plants consisted of two groups: herbivore treatment or undamaged control. In the first experiment, treatment emitters were damaged by saltmarsh caterpillars for 24 h and control emitter plants were left undamaged for the same amount of time (n = 10 boxes per treatment with 2 plants each). To induce damage on treatment emitters, four fifth-instar caterpillars were caged onto two individual leaves (two per leaf) using mesh bags (27 × 18 cm) to prevent the movement from emitter to receiver plants. Control plant leaves were bagged without insects. After 24 h of volatile exposure, we removed all emitter plants from their boxes. Each receiver was then challenged by caging four new third-instar saltmarsh caterpillars onto two separate leaves (two per bag) and allowing them to feed for 24 h. Once the 24 h ended, caterpillars were removed from receiver plants and a tissue sample was collected for later processing (~ 100 mg) from a leaf where feeding damage was present. The additional damaged leaf was cut from the base of the stem and scanned to quantify feeding damage (Fig. S3).

We repeated this experiment using either squash bug nymphs (n = 7 boxes per treatment with 2 plants each) or adult cucumber beetles (n = 8 boxes per treatment with 2 plants each). For squash bug trials, we caged four fifth-instar nymphs on treatment emitter plants for 48 h and exposed receiver plants to volatiles from these emitters. After exposures, emitter plants were removed, and then six third-instar nymphs were placed on two separate leaves (three per leaf) to damage receivers for 24 h (Fig. S3). After herbivore challenge, we removed nymphs to collect a tissue sample from one leaf and scan the other leaf for feeding damage as described above. For cucumber beetle trials, we caged five adult beetles on individual leaves of treatment emitters for 24 h. After exposures, emitters were removed from each box and receivers were damaged with six adult beetles on three separate leaves (two per leaf) for 24 h. Following herbivore challenge, beetles were removed, and tissue was collected from one leaf per receiver. The two additional damaged leaves were used for feeding damage quantification. Two separate trials were conducted for each insect species to confirm any trends seen in feeding damage or phytohormone analyses, although for squash bugs, phytohormone samples were only collected during the second trial.

### Leaf Area Damage Analysis

As an indicator of plant resistance, damaged leaves recovered from receiver plants were analyzed to calculate the amount of damage sustained from insect feeding (Karban et al. 2003; Helms et al. 2014). Chewing herbivore feeding damage was traced to enhance contrast for recognition by the ImageJ software (National Institutes of Health, Bethesda, USA). Comparatively, piercing-sucking damage from bugs was measured by the amount of discoloration present around a stylet puncture, which forms clearly visible lesions on damaged leaves due to the highly destructive style of Coreid feeding (Bonjour et al. 1991; Neal 1993). Discoloration around stylet punctures was also traced to enhance contrast and similarly analyzed using ImageJ. In the software, the area of leaf tissue removed by chewing herbivores or lesion area induced by piercing-sucking herbivores was measured and the total amount of damage per plant was calculated.

### Quantification of Phytohormones

To examine herbivore-induced plant defenses following volatile exposure, leaf tissue samples were collected from receiver plants after feeding damage and were used to quantify amounts of the defense hormones, jasmonic acid (JA) and salicylic acid (SA) present in the leaves (Helms et al. 2017; Harth et al. 2018). Samples were placed into liquid nitrogen after collection and stored in a -80 °C freezer until analysis. Extraction and quantification of JA and SA was replicated as previously described in Schmelz et al. (2003b, 2004). Plant hormones were extracted and derivatized to methyl esters, which were isolated using vapor-phase extraction. The compounds were analyzed by GC/CI-MS (Agilent Technologies, Santa Clara, USA) using isobutane and selected ion monitoring (SIM). We quantified amounts of jasmonic acid by adding 100 ng dihydro-JA to each sample as an internal standard and salicylic acid by adding 100 ng 2-Hydroxybenzoic Acid-d6 to each sample as an internal standard. The presence of these compounds was confirmed by comparing the retention times and spectra of the samples with standards of the compounds. To account for differences in herbivore feeding damage across individual receiver plants, we calculated the amount of each phytohormone per mass leaf tissue analyzed, per leaf area damaged (ng/g/cm^2^) (Schmelz et al. 2003a; Rodriguez-Saona et al. 2009).

### Statistical Analysis

Statistical analyses were performed using the software program R (R Version 3.6.1, R Core Team, 2019). Volatile data were analyzed by conducting permutational multivariate analysis of variance (PERMANOVA) for each herbivore species to quantify differences in volatile blends between herbivore-damaged and control plants (Oksanen et al. 2012; Clavijo Mccormick et al. 2014). Random forest analysis was used to identify compounds with the greatest contribution to variation between treatments (Ranganathan and Borges 2010; Ray et al. 2020). Non-metric multidimensional scaling (NMDS) ordinations were used to visualize volatile blend differences (Oksanen et al. 2012). Phytohormone data and herbivore feeding damage data were log- and square-root transformed, respectively, to meet assumptions of normality and analyzed using nested ANOVAs to account for non-independence between receivers from the same box.

## Results

### Three Herbivore Species Induce Distinct Blends of HIPVs from Squash Plants

**Fig. 1:**
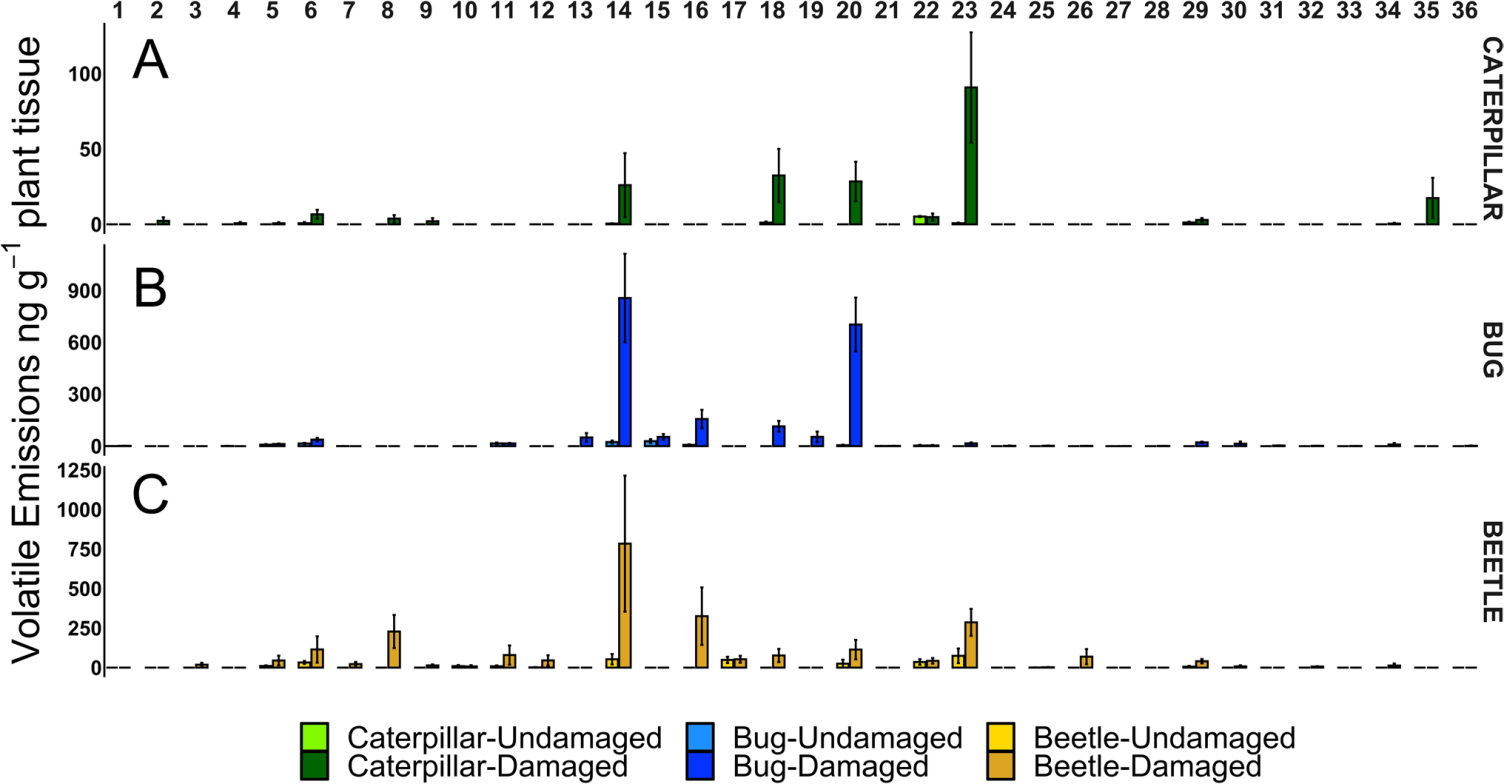
Feeding damage from different insect species induces distinct HIPV blends. **A**) Volatile compounds induced by saltmarsh caterpillar damage. **B**) Volatile compounds induced by squash bug damage. **C**) Volatile compounds induced by cucumber beetle damage. Compound numbers represent: 1. hexanal; 2. (*E*)-2-hexenal; 3. (*Z*)-3-hexen-1-ol; 4. anisole; 5. α-pinene; 6. benzaldehyde; 7. β-pinene; 8. 1-octen-3-ol; 9. (*Z*)-3-hexenyl acetate; 10. p-cymene; 11. limonene; 12. 6-camphenone; 13. (*Z*)-β-ocimene; 14. (*E*)-β-ocimene; 15. acetophenone; 16. (*E*)-4,8-dimethyl-1,3,7-nonatriene (DMNT); 17. nonanal; 18. linalool; 19. allo-ocimene; 20. methyl salicylate; 21. benzothiazole; 22. ethyl acetophenone; 23. indole; 24. **γ**-elemene; 25. α-gurjunene; 26. (*E*)- β-caryophyllene; 27. (*E*)-β-farnesene; 28. β-cubebene; 29. germacrene D; 30. (*E*)-α-farnesene; 31. germacrene B; 32. γ-muurolene; 33. δ-cadinene; 34. (*E*)-nerolidol; 35. farnesol; 36. methyl jasmonate. Means ± SE are presented

We collected foliar volatiles from squash plants with herbivory by either saltmarsh caterpillars, squash bug nymphs, or adult cucumber beetles or undamaged control plants. All three herbivore species induced significantly higher volatile production and distinct volatile blends compared to control plants (saltmarsh caterpillars PERMANOVA, *F*_1,9_ = 4.98, *p* = 0.01; squash bugs PERMANOVA, *F*_1,8_ = 10.22, *p* = 0.01; cucumber beetles PERMANOVA, *F*_1,9_ = 2.41, *p* = 0.03; Fig. S4), and the HIPV blends varied for the three herbivore species. Notably, HIPVs from saltmarsh caterpillar-damaged plants were less abundant and comprised fewer individual compounds compared to the other two herbivore species. We detected 14 compounds that were induced by saltmarsh caterpillar feeding relative to control plants (Fig. 1A, Table 1). Random Forest analysis revealed that (*E*)-β-ocimene, indole, linalool, and ethyl acetophenone were the dominant compounds differentiating the volatile blends from saltmarsh caterpillar-damaged and control plants (Fig. S5A). Caterpillar herbivory induced 3 unique compounds not detected in the other HIPV blends: (*E*)-2-hexanal, anisole, and farnesol. Herbivory by squash bug nymphs induced a total of 26 compounds (Fig. 1B, Table 1). The compounds methyl salicylate, (*Z*)-β-ocimene, (*E*)-4,8-dimethyl-1,3,7-nonatriene (DMNT), γ-muurolene, germacrene D, (*E*)-β-ocimene, indole, β-cubebene, (*E*)-α-farnesene, germacrene B, and allo-ocimene were the compounds of greatest importance in distinguishing the blends from squash bug-damaged and control plants (Fig. S5B). We detected a total of 22 compounds among HIPVs from plants with adult cucumber beetle herbivory (Fig. 1C, Table 1). The HIPV blend from plants with feeding by cucumber beetles was primarily influenced by DMNT, linalool, germacrene D, (*E*)-β-ocimene, 1-octen-3-ol, γ-muurolene, and indole (Fig. S5C). Overall, there were nine common compounds present across the HIPV blends for all three species. These included α-pinene, ethyl acetophenone, (*E*)-nerolidol, benzaldehyde, (*E*)-β-ocimene, linalool, methyl salicylate, indole, and germacrene D.

**Table 1:** Volatile compounds (ng g^−1^ plant tissue) induced by saltmarsh caterpillar, squash bug, and cucumber beetle feeding damage on squash plants. Volatiles in control blends that are not present in herbivore-damaged blends are not shown. Means ± SE are presented

Number	Volatile Compound	Herbivore Damage	Mean ± SE
1	hexanal	Caterpillar	0.00 ± 0.00
		Bug	1.85 ± 0.64
		Beetle	0.00 ± 0.00
2	(*E*)-2-hexenal	Caterpillar	2.32 ± 2.32
		Bug	0.00 ± 0.00
		Beetle	0.00 ± 0.00
3	(*Z*)-3-hexen-1-ol	Caterpillar	0.00 ± 0.00
		Bug	0.00 ± 0.00
		Beetle	18.14 ± 12.24
4	anisole	Caterpillar	0.74 ± 0.74
		Bug	0.00 ± 0.00
		Beetle	0.00 ± 0.00
5	α-pinene	Caterpillar	0.70 ± 0.70
		Bug	12.46 ± 2.31
		Beetle	44.81 ± 30.83
6	benzaldehyde	Caterpillar	6.67 ± 2.95
		Bug	37.86 ± 10.12
		Beetle	114.60 ± 83.13
7	β-pinene	Caterpillar	0.00 ± 0.00
		Bug	0.00 ± 0.00
		Beetle	22.10 ± 13.61
8	1-octen-3-ol	Caterpillar	3.81 ± 2.34
		Bug	0.00 ± 0.00
		Beetle	228.07 ± 104.91
9	(*Z*)-3-hexenyl acetate	Caterpillar	2.06 ± 2.06
		Bug	0.00 ± 0.00
		Beetle	13.39 ± 6.98
10	*p*-cymene	Caterpillar	0.00 ± 0.00
		Bug	0.00 ± 0.00
		Beetle	7.51 ± 7.51
11	limonene	Caterpillar	0.00 ± 0.00
		Bug	15.48 ± 3.29
		Beetle	79.26 ± 60.63
12	6-camphenone	Caterpillar	0.00 ± 0.00
		Bug	0.00 ± 0.00
		Beetle	45.47 ± 32.63
13	(*Z*)-β-ocimene	Caterpillar	0.00 ± 0.00
		Bug	50.40 ± 25.10
		Beetle	0.00 ± 0.00
14	(*E*)-β-ocimene	Caterpillar	26.00 ± 21.23
		Bug	856.78 ± 256.21
		Beetle	784.09 ± 430.32
15	acetophenone	Caterpillar	0.00 ± 0.00
		Bug	53.59 ± 16.51
		Beetle	0.00 ± 0.00
16	(*E*)-4,8-dimethyl-1,3,7-nonatriene (DMNT)	Caterpillar	0.00 ± 0.00
		Bug	157.37 ± 53.03
		Beetle	325.33 ± 181.98
17	nonanal	Caterpillar	0.00 ± 0.00
		Bug	0.00 ± 0.00
		Beetle	52.77 ± 21.62
18	linalool	Caterpillar	32.41 ± 17.67
		Bug	114.65 ± 31.48
		Beetle	76.63 ± 41.74
19	allo-ocimene	Caterpillar	0.00 ± 0.00
		Bug	53.89 ± 30.54
		Beetle	0.00 ± 0.00
20	methyl salicylate	Caterpillar	28.48 ± 13.04
		Bug	702.95 ± 156.29
		Beetle	113.93 ± 61.56
21	benzothiazole	Caterpillar	0.00 ± 0.00
		Bug	1.46 ± 0.71
		Beetle	0.00 ± 0.00
22	ethyl acetophenone	Caterpillar	4.79 ± 2.39
		Bug	4.21 ± 2.22
		Beetle	42.71 ± 17.47
23	indole	Caterpillar	90.84 ± 36.56
		Bug	15.91 ± 5.81
		Beetle	286.28 ± 85.47
24	**γ**-elemene	Caterpillar	0.00 ± 0.00
		Bug	1.83 ± 1.83
		Beetle	0.00 ± 0.00
25	α-gurjunene	Caterpillar	0.00 ± 0.00
		Bug	2.29 ± 1.41
		Beetle	2.20 ± 1.15
26	(*E*)-β-caryophyllene	Caterpillar	0.00 ± 0.00
		Bug	1.36 ± 0.37
		Beetle	69.01 ± 48.04
27	(*E*)-β-farnesene	Caterpillar	0.00 ± 0.00
		Bug	0.38 ± 0.16
		Beetle	0.00 ± 0.00
28	β-cubebene	Caterpillar	0.00 ± 0.00
		Bug	1.23 ± 0.22
		Beetle	0.00 ± 0.00
29	germacrene D	Caterpillar	2.94 ± 1.21
		Bug	21.96 ± 4.03
		Beetle	40.08 ± 13.96
30	(*E*)-α-farnesene	Caterpillar	0.00 ± 0.00
		Bug	14.06 ± 12.45
		Beetle	7.74 ± 6.31
31	germacrene B	Caterpillar	0.00 ± 0.00
		Bug	3.93 ± 0.61
		Beetle	0.00 ± 0.00
32	**γ**-muurolene	Caterpillar	0.00 ± 0.00
		Bug	1.93 ± 0.47
		Beetle	6.60 ± 1.74
33	δ-cadinene	Caterpillar	0.00 ± 0.00
		Bug	0.82 ± 0.39
		Beetle	0.00 ± 0.00
34	(*E*)-nerolidol	Caterpillar	0.49 ± 0.49
		Bug	9.67 ± 8.02
		Beetle	12.88 ± 12.88
35	farnesol	Caterpillar	17.41 ± 13.46
		Bug	0.00 ± 0.00
		Beetle	0.00 ± 0.00
36	methyl jasmonate	Caterpillar	0.00 ± 0.00
		Bug	2.05 ± 1.75
		Beetle	0.00 ± 0.00
	Total	Caterpillar Bug Beetle	219.66 ± 92.21 1983.01 ± 488.08 2393.61 ± 1096.75

### HIPVs from Plants Damaged by Different Herbivore Species Have Contrasting Effects on Plant Resistance to Herbivory

**Fig. 2:**
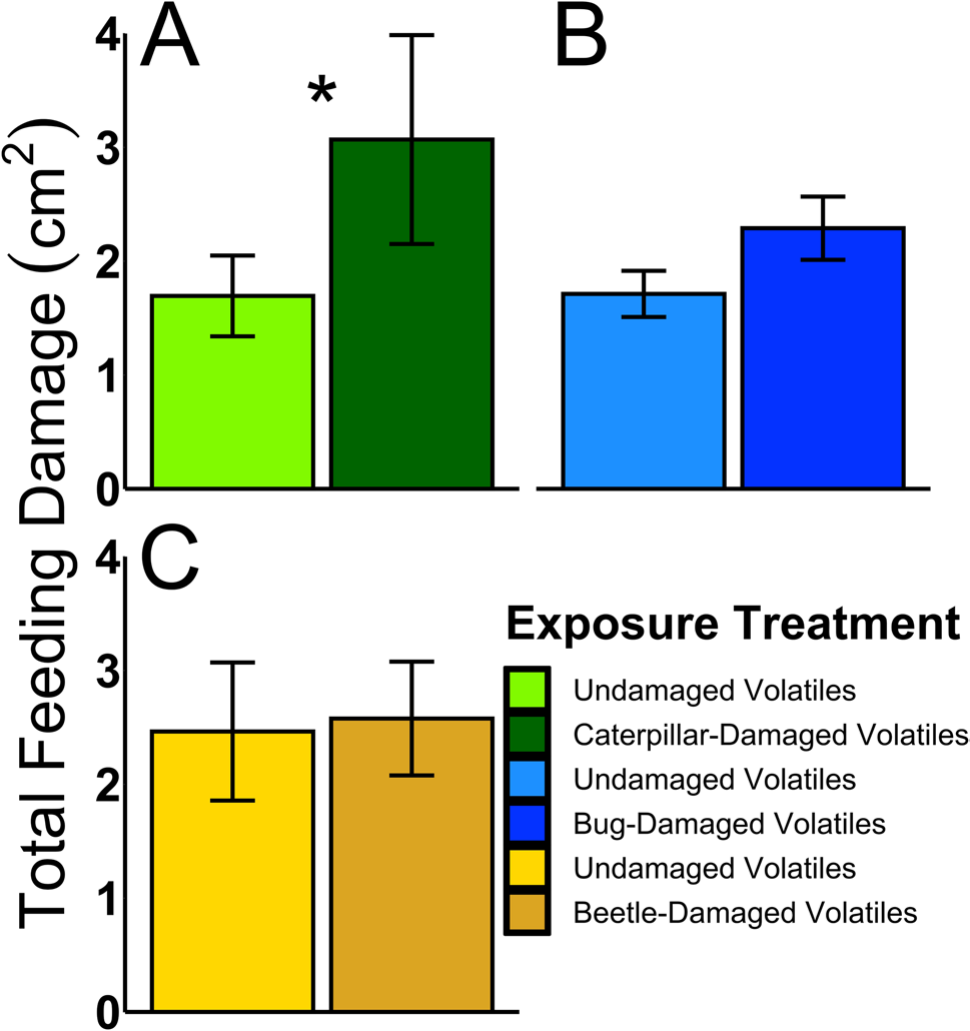
Exposing squash plants to volatiles from neighboring plants damaged by different herbivore species had contrasting effects on plant resistance to herbivores. A) Exposure to HIPVs significantly increased feeding damage by saltmarsh caterpillars on neighboring plants. B) Squash bug nymphs inflicted a similar amount of leaf damage on HIPV-exposed and control receiver plants. C) Adult cucumber beetles consumed a similar amount of leaf tissue on HIPV-exposed and control receiver plants. (*p ≤ 0.05). Means ± SE are presented

To evaluate whether exposing squash plants to volatiles from neighboring plants with herbivory by different herbivore species influenced their resistance, we conducted laboratory feeding bioassays. Contrary to our predictions, we found that squash plants exposed to volatiles from other plants with saltmarsh caterpillar herbivory were more susceptible to herbivore feeding damage, with caterpillars consuming significantly more leaf tissue on HIPV-exposed receiver plants compared to control plants (ANOVA, *F*_1,20_ = 5.11, *p* = 0.03, Fig. 2A). In contrast, we found no evidence of either suppressed or enhanced resistance in plants exposed to squash bug-induced volatiles, as squash bug nymphs fed similarly on both HIPV-exposed and control plants (ANOVA, *F*_1,14_ = 1.71, *p* = 0.21, Fig. 2B). Exposing receiver plants to cucumber beetle-induced volatiles also had no effect on plant resistance to beetles compared to control plants. Adult cucumber beetles consumed similar amounts of leaf tissue on plants exposed to HIPVs or volatiles from undamaged plants (ANOVA, *F*_1,16_ = 0.55, *p* = 0.47, Fig. 2C). This indicates that exposure to HIPVs induced by different herbivores had contrasting effects on neighboring plant resistance to herbivory.

### Plant Exposure to HIPVs from Different Herbivore Species Has Contrasting Effects on Plant Defenses

**Fig. 3:**
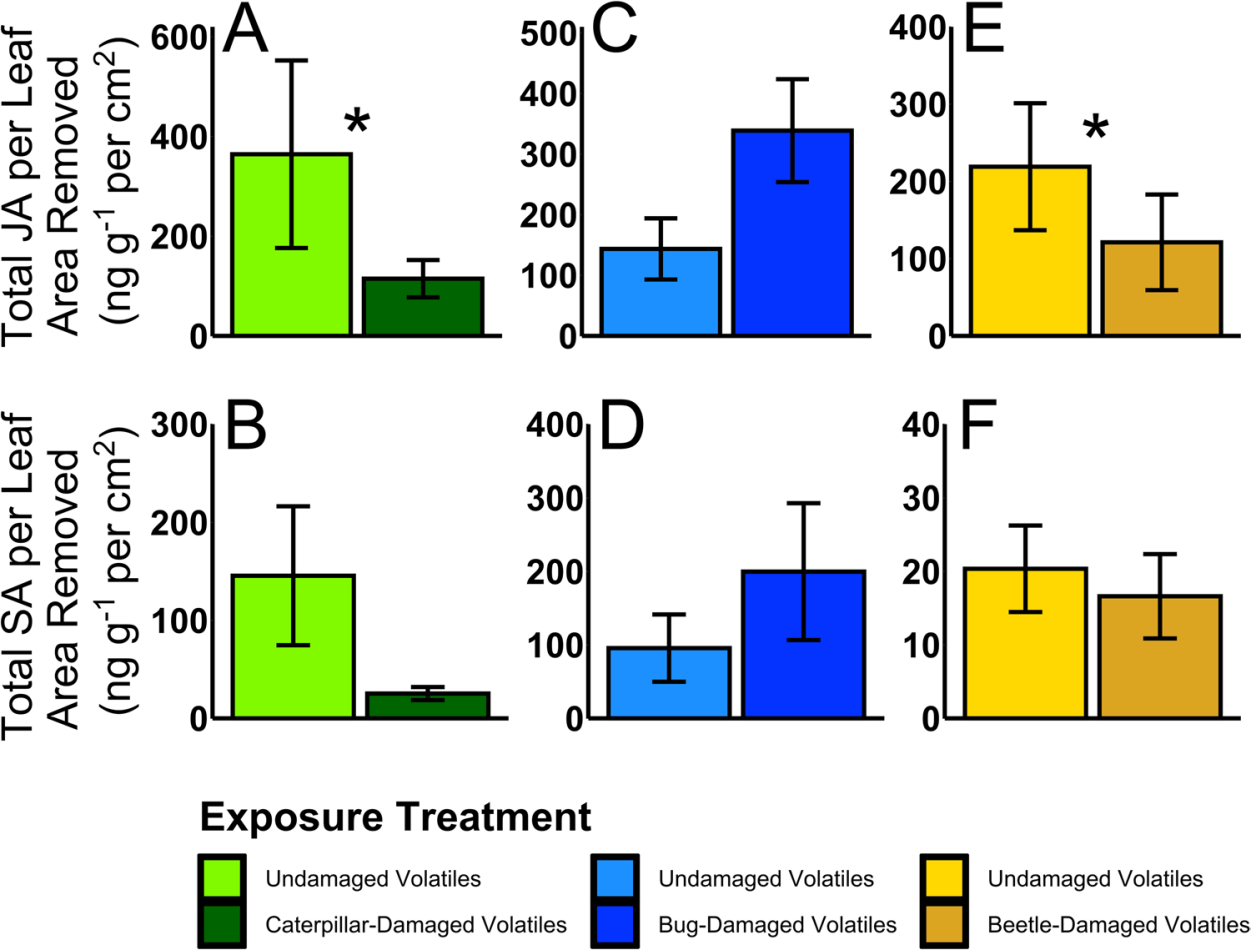
Plant exposure to HIPVs induced by different herbivore species had contrasting effects on plant defense responses. A-B) Plant exposure to saltmarsh caterpillar-induced HIPVs reduced induction of JA but not SA. C-D) Squash bug nymphs induced a similar amount of JA and SA on plants exposed to HIPVs or undamaged volatiles. E–F) Plant exposure to cucumber beetle-induced HIPVs suppressed JA induction but did not alter induced levels of SA. Means ± SE are presented

To further evaluate how HIPVs from plants with herbivory by different herbivore species influence squash defense responses, we quantified levels of the key defense-related phytohormones, jasmonic acid (JA) and salicylic acid (SA), in HIPV-exposed and unexposed plants after feeding by each herbivore species. Plants exposed to HIPVs from saltmarsh caterpillar*-*damaged emitters induced significantly less JA (ANOVA, *F*_1,19_ = 5.47, *p* = 0.03, Fig. 3A) but no difference in SA (ANOVA, *F*_1,20_ = 2.26, *p* = 0.15, Fig. 3B) compared to unexposed control plants. This indicates that saltmarsh caterpillar-induced HIPVs compromised defense responses in receiver plants, rendering them more susceptible to subsequent herbivore damage. In contrast, receiver squash plants exposed to HIPVs from emitters with squash bug herbivory had no differences in JA (ANOVA, *F*_1,4_ = 3.20, *p* = 0.15, Fig. 3C) or SA (ANOVA, *F*_1,4_ = 0.61, *p* = 0.48, Fig. 3D) induced by subsequent squash bug herbivory relative to control plants. Receiver plants exposed to cucumber beetle HIPVs had significantly lower induced JA compared to plants exposed to control volatiles (ANOVA, *F*_1,16_ = 9.92, *p* = 0.01, Fig. 3E), while induced SA levels in both undamaged-exposed and HIPV-exposed receivers were not different (ANOVA, *F*_1,16_. = 0.49, *p* = 0.49, Fig. 3F).

## Discussion

Herbivore species identity (De Moraes et al. 2001), feeding guild (Chung and Felton 2011), and host range (Paudel Timilsena et al. 2020) can drive qualitative and quantitative differences in HIPV blends. It is well-documented that herbivore species-specific HIPVs can selectively attract different natural enemies to attack particular herbivore species (De Moraes et al. 1998; Clavijo McCormick et al. 2012), suggesting a high level of specificity in natural enemy recruitment for indirect plant defense. Relative to natural enemy attraction, our understanding of whether specific HIPVs elicit different priming responses in neighboring plants lags behind and plant priming specificity remains an open question. Although recent evidence points towards volatile cues priming non-specific defense responses (Helms et al. 2017; Paudel Timilsena et al. 2020), other studies highlight specificity in interplant communication (Choh et al. 2013; Karban et al. 2013; Moreira et al. 2018). Our study investigated herbivore species-specificity of plant defense priming following exposure to three different HIPV blends from squash plants. Contrary to our predictions, we found no evidence of HIPV-mediated priming for any of the three herbivore species tested, although each herbivore species induced unique HIPV blends (Fig. 1) and defense responses in neighboring plants. We determined exposure to specialist cucumber beetle-induced or squash bug-induced HIPVs did not affect neighboring plant resistance against these herbivores (Figs. 2B-C). In contrast, exposure to generalist saltmarsh caterpillar-induced HIPVs suppressed defenses in neighboring squash plants (Fig. 3A), increasing their susceptibility to caterpillar herbivory (Fig. 2A). Our findings reveal a new example of volatile-mediated defense suppression by a polyphagous, generalist herbivore and provide additional insights into the role of HIPVs in modulating plant responses to insect herbivores.

In addition to feeding guild, herbivore host-plant specialization can also influence plant defense (Ali and Agrawal 2012). Evidence for differences between specialists and generalists in HIPV production remains mixed, with a recent meta-analysis supporting more HIPV production following specialist herbivory (Rowen and Kaplan 2016) and other studies documenting greater HIPV production in response to generalist herbivores (Sobhy et al. 2017; Danner et al. 2018). Despite representing different feeding guilds, the two specialist herbivore species in our study induced the greatest number and highest amounts of compounds in their HIPV blends (Fig. 1, Table 1). We expected squash bug herbivory to induce lower levels of HIPVs, as piercing-sucking herbivores generally cause less damage to plant tissues than chewing herbivores. However, squash bug feeding inflicts relatively high amounts of damage compared to other piercing-sucking herbivores (Bonjour et al. 1991; Neal 1993), which could help explain the enhanced HIPV production relative to generalist caterpillars. Moreira et al. (2018) documented specialist and generalist aphid herbivory induced species-specific HIPV emissions from the shrub *Baccharis salicifolia*, with priming of neighboring plants only occurring following exposure to HIPVs induced by the subsequently attacking herbivore. In our study, plant exposure to beetle-induced HIPVs weakened induction of JA (Fig. 3E), however, this did not increase plant susceptibility to the beetles (Fig. 2C). A possible explanation is that striped cucumber beetles are specialists on cucurbit plants and adapted to tolerate squash plant defenses (Tallamy and Gorski 1997; Brzozowski et al. 2020). We observed a trend toward higher induction of JA in plants exposed to HIPVs induced by squash bugs (Fig. 3C), but this slight increase did not confer enhanced resistance against the herbivores (Fig. 2B), again, possibly because they are specialists adapted to cucurbit defenses (Mauck et al. 2010b; Brzozowski et al. 2021).

In contrast to the specialist herbivores, generalist saltmarsh caterpillars induced lower levels of volatiles and fewer overall compounds (Fig. 1, Table 1). As chewing herbivores inflicting greater tissue damage than the other two species, we expected saltmarsh caterpillars would induce the greatest production of volatile compounds (Schmelz et al. 2003a). It is possible low volatile induction is an adaptive strategy by the caterpillars to avoid HIPV-mediated direct and indirect plant defenses. HIPV emissions from squash directly defend against two generalist caterpillar species (*Trichoplusia ni* and *Spodoptera exigua*) (Brzozowski et al. 2019), suggesting suppressed volatile induction may benefit saltmarsh caterpillars feeding on squash. In terms of indirect defense, because saltmarsh caterpillars are attacked by several parasitoid species in nature (Singer et al. 2004), reducing HIPV emissions could allow caterpillars to ‘hide’ from parasitoids using HIPVs as host-location cues. Notably, another generalist caterpillar species (*Helicoverpa zea*) suppresses HIPV production when feeding on tomato plants. An effector molecule, glucose oxidase (GOX), in the caterpillar saliva induces stomatal closure that limits the release of HIPVs (Lin et al. 2021). Future research should characterize the presence and activity of effector molecules in saltmarsh caterpillar oral secretions to determine their role in modulating HIPVs and interplant communication (Felton and Tumlinson 2008; Acevedo et al. 2019). It is also important to note that the density and life stage of caterpillars used in this study may have affected the results we observed. Saltmarsh caterpillars feed gregariously during the first three instars, however, by the fifth instar—as caterpillars are larger and consume more leaf tissue per individual—they typically begin foraging alone (Singer et al. 2004). In general, as herbivore density and/or plant damage increases, volatile blends change quantitatively but not qualitatively (Shiojiri et al. 2010; Cai et al. 2014), indicating emitter plants could have stronger HIPV production with more caterpillars or damage. Plant ontogeny can also affect defense induction and HIPV emission, as investments in defense often shift throughout plant development (Boege and Marquis 2005; Mertens et al. 2021).

A surprising finding from our study was that exposure to saltmarsh caterpillar-induced HIPVs suppressed defense responses in neighboring plants (Fig. 3A-B), enhancing their susceptibility to caterpillar herbivory (Fig. 2A). The majority of studies on plant responses to HIPVs have reported increased resistance to herbivory, highlighting interplant communication as a potential strategy for plants to better predict and defend against herbivore attack (Karban et al. 2014). In contrast, HIPV-mediated defense suppression has only been reported for a few plant–herbivore species combinations (Pearse et al. 2012; Li and Blande 2015; Zhang et al. 2019), and, more recently, plant exposure to an insect pheromone was also shown to suppress defenses (Brosset et al. 2021). Although the exact mechanisms underlying increased susceptibility in these studies have not been fully elucidated, Zhang et al. (2019) found HIPVs induced by generalist whiteflies on tomato enhanced SA but suppressed JA through phytohormone crosstalk, ultimately resulting in enhanced whitefly performance on neighboring plants. Our findings indicate that JA-dependent defenses were suppressed in squash plants exposed to HIPVs induced by saltmarsh caterpillar herbivory, with a trend toward lower SA defenses (Fig. 3A, B), indicating the possibility for a previously undescribed suppression mechanism acting on both pathways. It is notable that the two major volatile compounds contributing to caterpillar-induced HIPVs—indole and (*E*)-β-ocimene—have both been shown to prime plants in other systems (Muroi et al. 2011; Cascone et al. 2015; Erb et al. 2015), and (*E*)-β-ocimene primed both SA- and JA- dependent defenses in cabbage (Kang et al. 2018). Intriguingly, indole can inhibit the phytohormone auxin, indole-acetic-acid (IAA), in *Arabidopsis thaliana* (Bailly et al. 2014). Since IAA acts synergistically with JA in *Nicotiana attenuata* to induce defenses (Machado et al. 2016), it has been previously proposed that indole suppression of IAA could also reduce JA, thereby suppressing plant defense against herbivory (Erb 2018). However, indole and (*E*)-β-ocimene were not unique to the caterpillar-HIPV blends, which may suggest that other compounds or the full caterpillar-HIPV blend are necessary to elicit a response (Moreira et al. 2018). We identified 3 compounds in the saltmarsh caterpillar-induced HIPVs not present for the other two species, (*E*)-2-hexanal, anisole, and farnesol, although these compounds were not detected in all caterpillar-damaged plant volatile collections. Future studies should also work to determine if specific compounds in the HIPV blends were responsible for suppressing plant defenses.

Finally, an outstanding question remains as to whether defense suppression benefits saltmarsh caterpillars, or possibly emitter plants. Saltmarsh caterpillars are a highly polyphagous and mobile species capable of detoxifying or sequestering plant toxins like pyrrolizidine alkaloids and iridoid glycosides (Hartmann et al. 2005; Lampert and Bowers 2010), facilitating feeding on a broad range of host plants throughout their development (Singer et al. 2004). Based on our findings, it is possible that caterpillars actively manipulate the defenses of neighboring plants through HIPVs to make them more palatable as future food sources. However, enhanced caterpillar feeding damage on plants exposed to caterpillar-induced HIPVs could also indicate compensatory feeding, as it is possible HIPV exposure resulted in nutritional changes in squash receiver plants. Characterizing specific defensive traits and plant nutrients in HIPV-exposed squash plants will help to identify the defensive mechanisms responsible for this increased susceptibility. Alternatively, following herbivore attack, plants can use different strategies to compete with neighboring plants and enhance their own fitness (Backmann et al. 2019). Volatile communication can shape competition between plants (Effah et al. 2019), suggesting HIPV emitters may sabotage neighboring receivers through deceitful communication, inducing vulnerability in receivers to herbivore attack. However, it is worth noting that these findings represent a short snapshot of an ecological interaction between plants and herbivores, and the timing and duration of HIPV exposure could influence receiver responses. Despite these caveats, defense suppression in neighboring receivers could also provide alternative explanations for how HIPV-mediated interplant communication evolved. Current theory predicts HIPV communication most likely evolved to overcome within-plant vascular constraints and aid in systemic activation of defenses within an attacked plant (Heil and Ton 2008; Heil and Karban 2010). Under this definition, neighboring receiver plant detection of HIPVs represents a case of ‘eavesdropping’ rather than intended communication from emitters (Heil and Karban 2010). However, defense suppression in neighboring receivers begs the question if emitters in this system evolved to decrease the fitness of neighboring plants, thereby possibly enhancing their own fitness. Strong competition between plants is predicted to drive HIPV-mediated defense suppression in neighbors (Pearse et al. 2012), although crop breeding often selects for reduced competition between conspecifics. Future studies in this system should quantify the effects of HIPV exposure on caterpillar and plant performance and fitness to determine if either organism benefits ecologically or evolutionary from this defense suppression.

## Supplementary Information

Below is the link to the electronic supplementary material.

Supplementary file1 (DOCX 10638 KB)

## Data Availability

Data will be available from the Dryad Digital Repository following acceptance for publication.
