# Supplementary material for: Defense Suppression through Interplant Communication Depends on the Attacking Herbivore Species
Source: J Chem Ecol. 2021 Sep 20;47(12):1049–61. doi: 10.1007/s10886-021-01314-6 (PMC8642252; doi:10.1007/s10886-021-01314-6)
Supplement: Supplementary file 1 — Supplementary file1 (DOCX 10638 KB) [file 10886_2021_1314_MOESM1_ESM.docx]

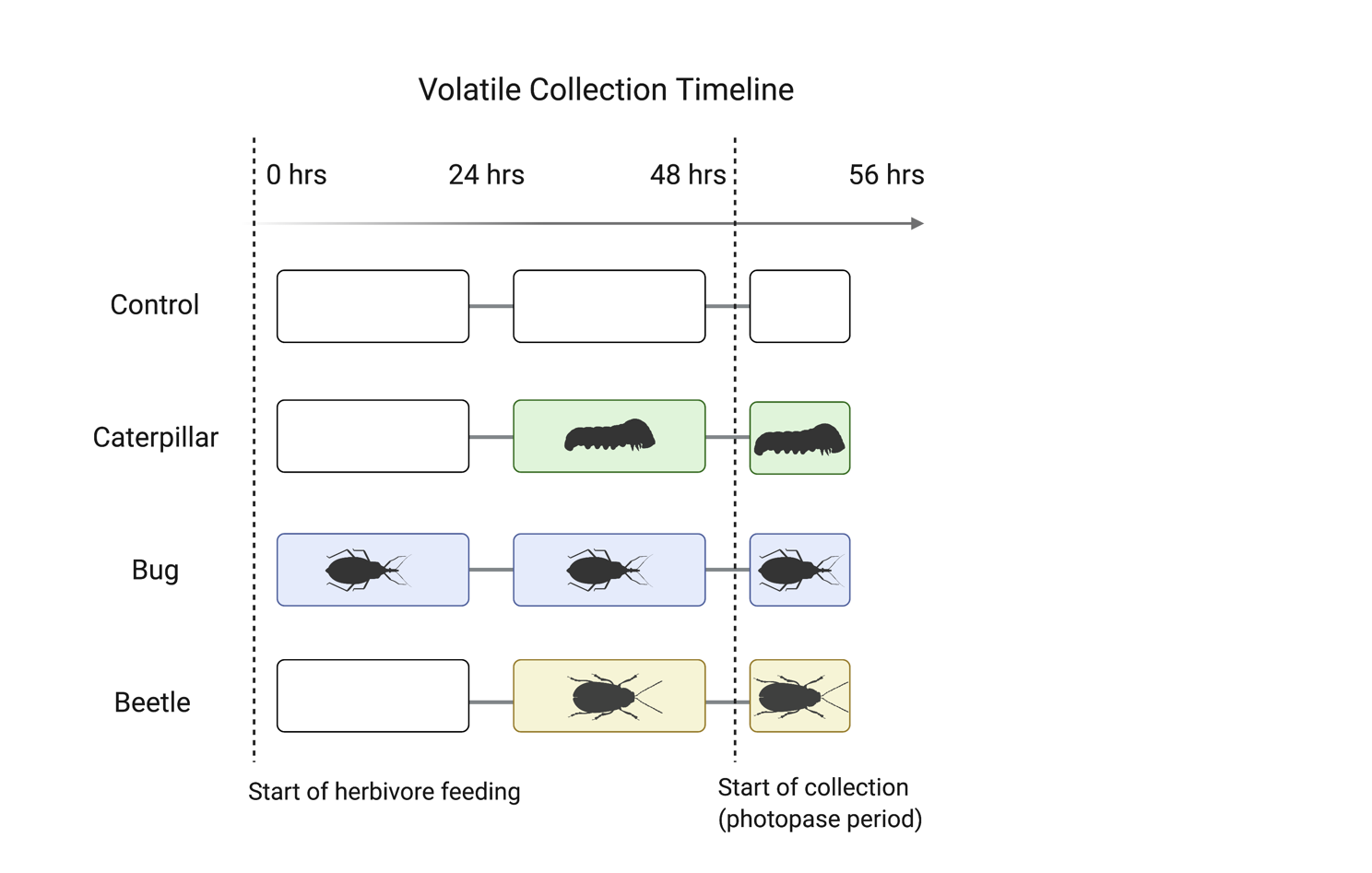
**Supplemental Materials**

**Figure S1.** Timeline for volatile collections from control and herbivore-damaged squash plants. Insect silhouettes indicate herbivores feeding.


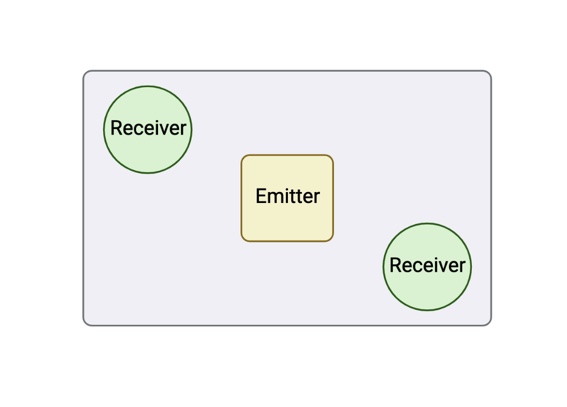


**Figure S2.** Diagram of the volatile exposure and herbivore challenge experimental set up. One emitter plant was surrounded by two receiver plants during the exposure stage, and was then removed after 24 hours.


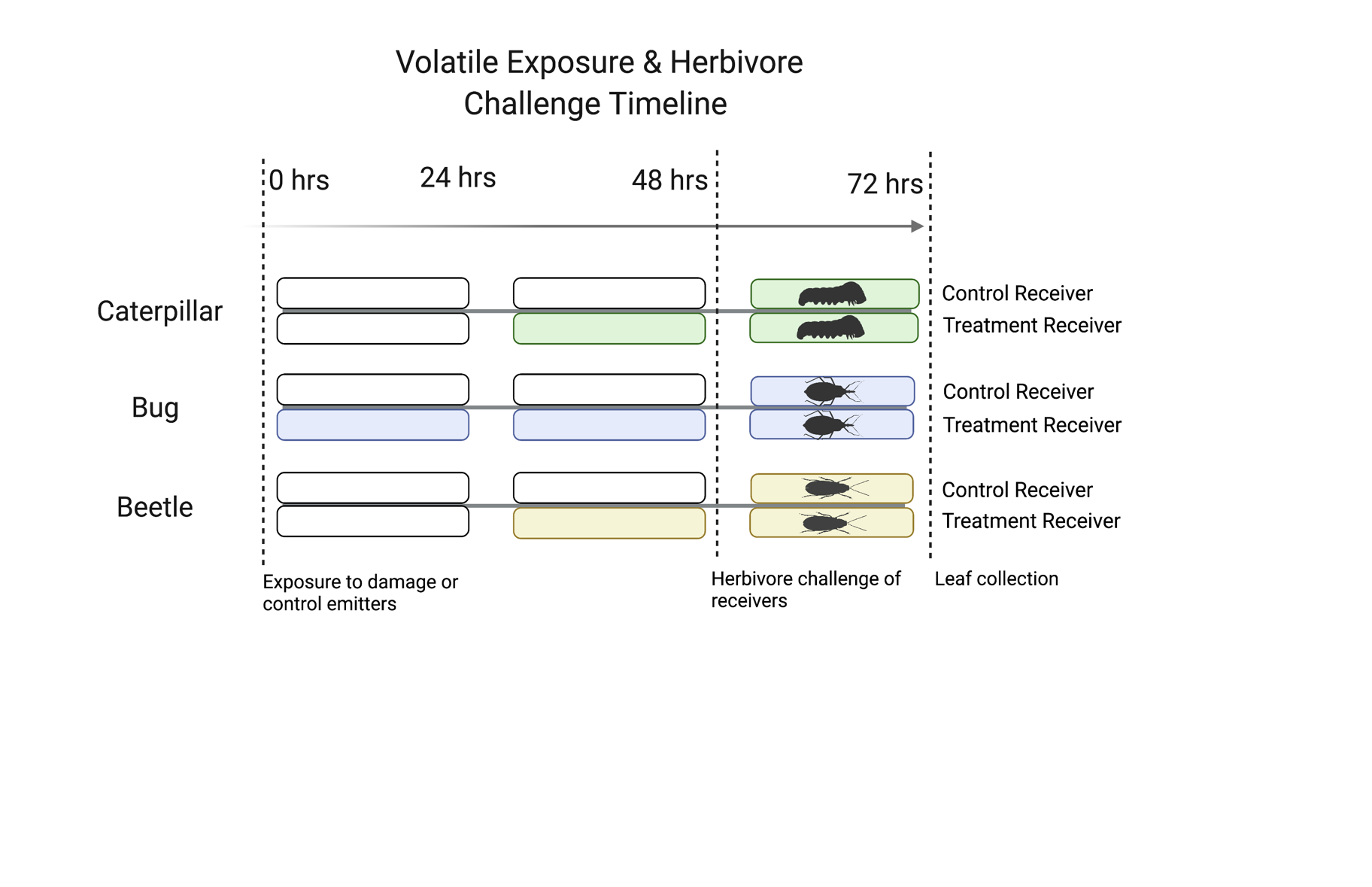


**Figure S3.** Timeline of volatile exposure and herbivore challenge experiment for receiver plants. Colored bars represent HIPV exposure treatments (green=caterpillars, blue=bugs, yellow=beetles), white bars are undamaged controls. Insect silhouettes indicate herbivores feeding on receivers.


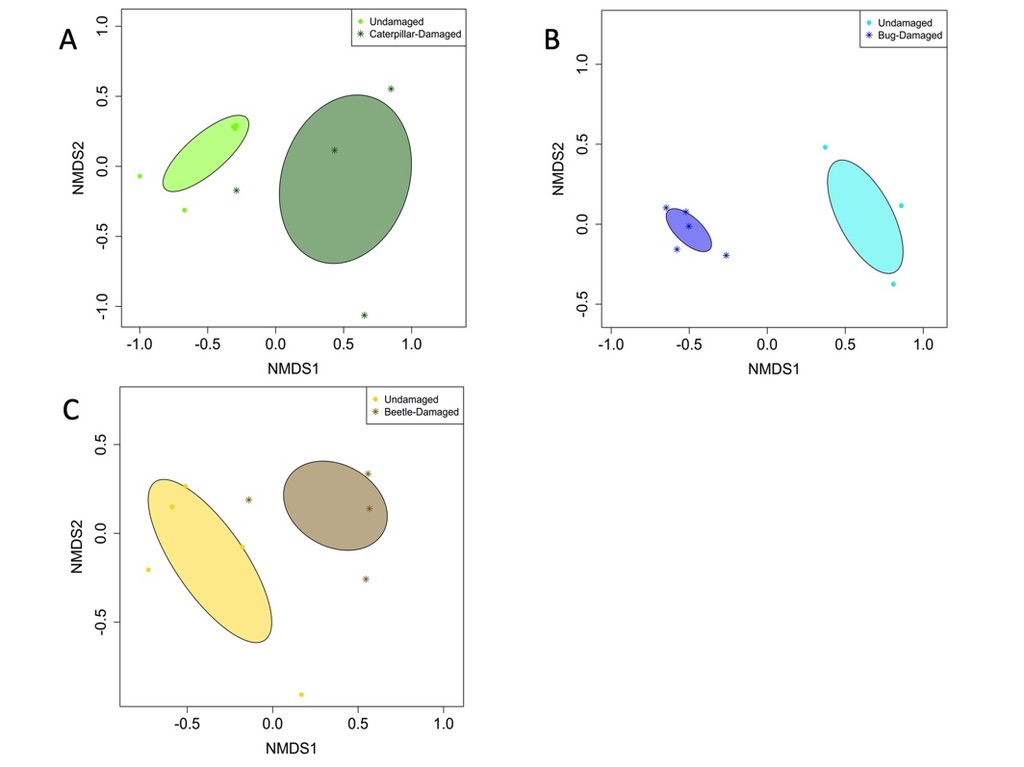


**Figure S4**. NMDS plots representing the volatile blends of squash plants damaged by herbivory or undamaged control plants. Each herbivore species (A. saltmarsh caterpillars, B. squash bugs, C. cucumber beetles) produced significantly higher amounts of volatiles compared to undamaged plants, each with differing volatile blends. Ellipses represent the standard deviation around each group’s centroid.


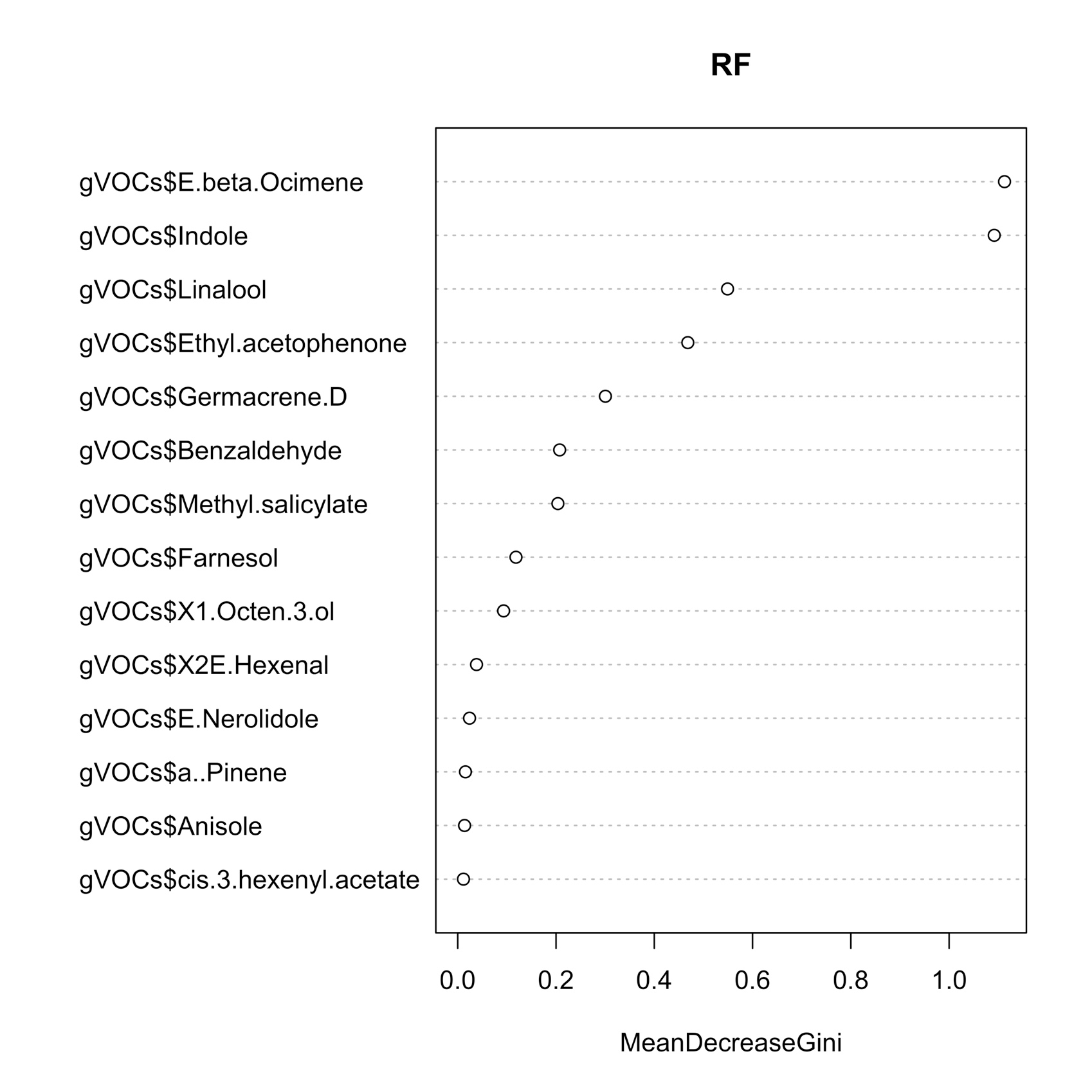


A


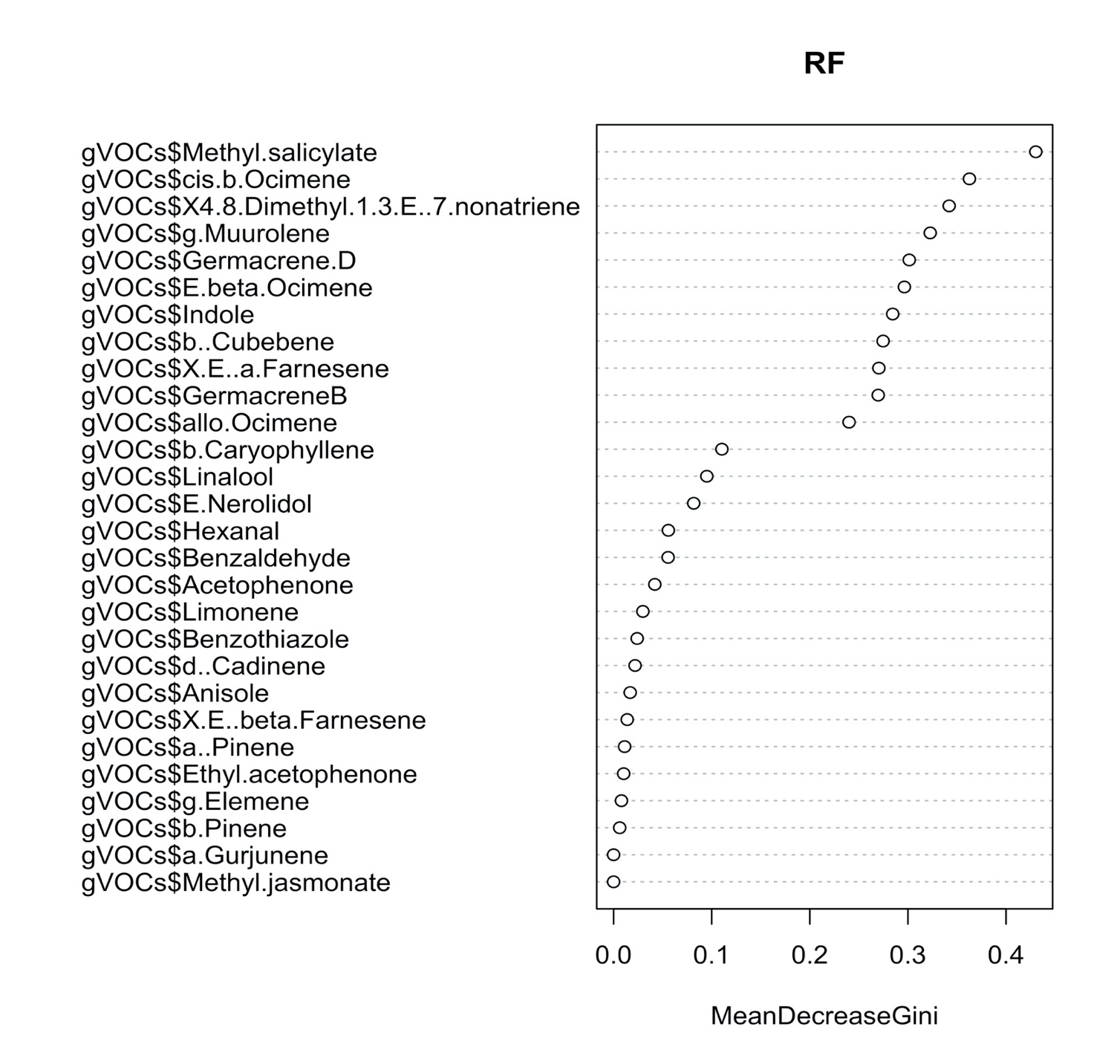


B


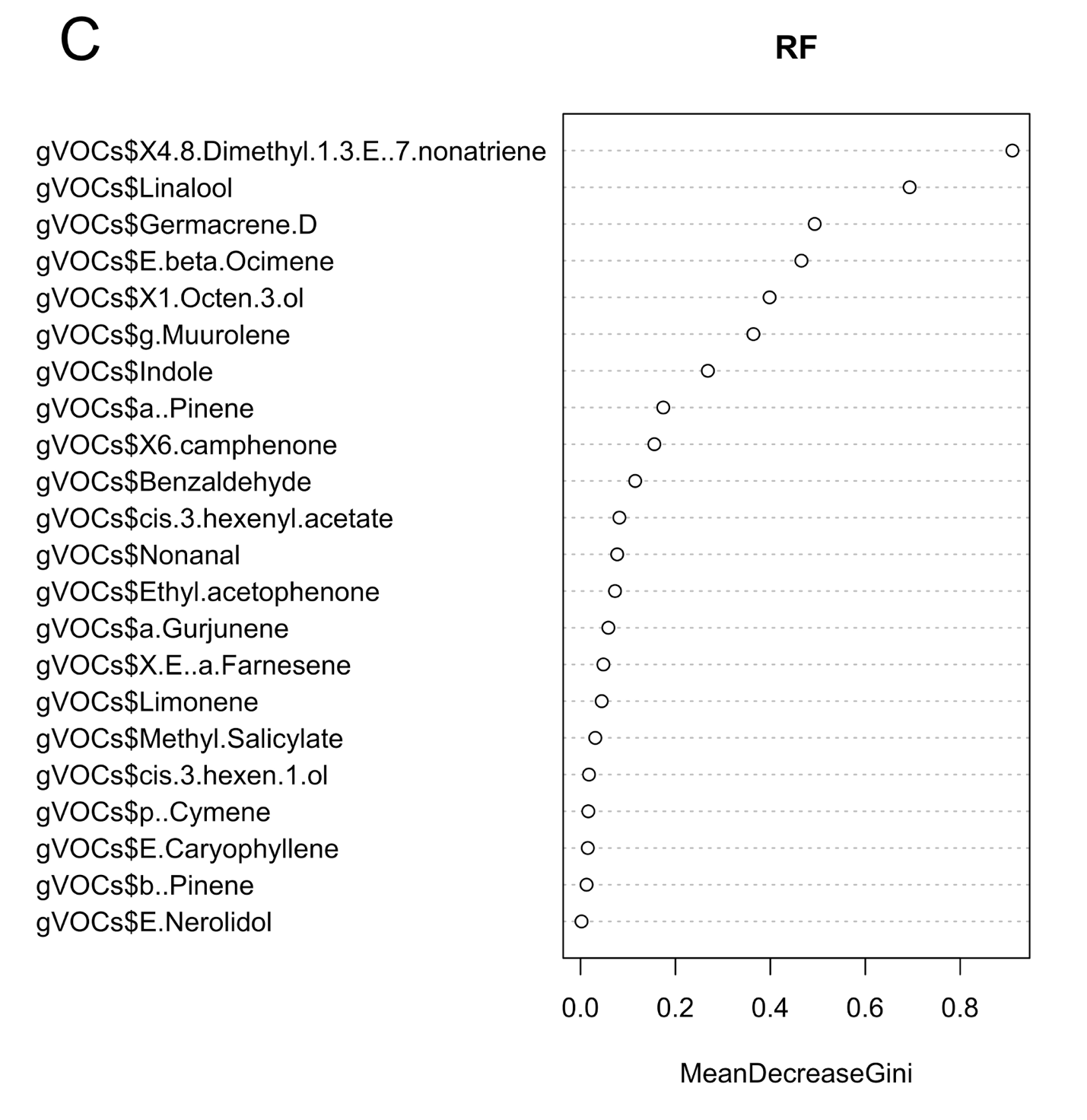


**Figure S5**. Random Forest Analyses for the volatile blends of squash plants damaged by herbivory or undamaged control plants. A) saltmarsh caterpillars, B) squash bugs, C) cucumber beetles) The Gini scores indicate individual compounds with the greatest contribution to differentiating between the herbivore-induced and control volatile blends.
